# The Role of Continuous Peripheral Nerve Blocks

**DOI:** 10.1155/2012/560879

**Published:** 2012-06-18

**Authors:** José Aguirre, Alicia Del Moral, Irina Cobo, Alain Borgeat, Stephan Blumenthal

**Affiliations:** ^1^Division of Anesthesiology, Balgrist University Hospital, 8008 Zurich, Switzerland; ^2^Department of Anesthesiology, General University Hospital of Valencia, 46014 Valencia, Spain; ^3^Department of Anesthesiology, Triemli Hospital, 8063 Zurich, Switzerland

## Abstract

A continuous peripheral nerve block (cPNB) is provided in the hospital and ambulatory setting. The most common use of CPNBs is in the peri- and postoperative period but different indications have been described like the treatment of chronic pain such as cancer-induced pain, complex regional pain syndrome or phantom limb pain. The documented benefits strongly depend on the analgesia quality and include decreasing baseline/dynamic pain, reducing additional analgesic requirements, decrease of postoperative joint inflammation and inflammatory markers, sleep disturbances and opioid-related side effects, increase of patient satisfaction and ambulation/functioning improvement, an accelerated resumption of passive joint range-of-motion, reducing time until discharge readiness, decrease in blood loss/blood transfusions, potential reduction of the incidence of postsurgical chronic pain and reduction of costs. Evidence deriving from randomized controlled trials suggests that in some situations there are also prolonged benefits of regional anesthesia after catheter removal in addition to the immediate postoperative effects. Unfortunately, there are only few data demonstrating benefits after catheter removal and the evidence of medium- or long-term improvements in health-related quality of life measures is still lacking. This review will give an overview of the advantages and adverse effects of cPNBs.

## 1. Background

Since its first description in 1946, cPNB has evolved from a case report of a needle inserted through a cork taped to a patient's chest to a wide-spread and validated analgesic technique in the postoperative setting [1]. The earliest reports of cPNB focused on prolonging intraoperative surgical anesthesia and the treatment of intractable hiccups [1–3]. The indication for cPNB has evolved since then, and many indications have been described in the literature: treatment of vasospasm induced by Raynaud disease [4]; induction of sympathectomy and vasodilation for improvement of blood flow after vascular surgery/trauma [5, 6], replantation or limb salvage [7, 8]; treatment of peripheral embolism [9, 10]; analgesia in the setting of trauma [11]; treatment of chronic pain syndrome such as trigeminal neuralgia [12], complex regional pain syndrome [13], terminal cancer pain, [14], and phantom limb pain [15, 16]. Independently of these indications, the majority of publications dealing with cPNB focus on postsurgical pain treatment, where evidence supports the concept that regional anesthesia and analgesia offers superior pain relief to systemic opioid analgesia following major surgery [17]. However, postsurgical pain is the only indication which has been validated using randomized controlled trials (RCTs) [18–22]. Compared with opioid analgesics cPNBs provide superior analgesia with a lower incidence of opioid-induced side effects like nausea, vomiting, pruritus, and sedation [21]. Moreover, in a meta-analysis Rodgers et al. suggested that mortality associated with major surgery was reduced by 30% when central regional anesthesia was used combined or not with general anesthesia [23]. On the other hand, a Cochrane review showed no impact of regional anesthesia compared to general anesthesia on mortality after hip fracture. Only the acute postoperative confusion was reduced after regional anesthesia [24]. However, for continuous peripheral regional anesthesia these findings cannot be extrapolated. Though, continuous peripheral regional anesthesia offers improved functional outcomes after extremity surgery at least for a short term period (up to 6 months) [25–27]. 

Despite this evidence of value, the hypothesis that regional anesthesia has an overall beneficial and long-lasting effect on patient outcome following surgery still remains difficult to prove and has been challenged, especially in times with reduced resources. For more than 30 years, regional anesthesia has challenged anesthesiologists to determine whether it offers real benefits over other types of anesthesia, such as preserving cognitive function after major surgery compared to general anesthesia, improving long-term joint function and rehabilitation leading to earlier return to work, reducing costs, reducing the need for blood transfusions and increasing patient-reported outcomes such as satisfaction, quality of life, and quality of recovery.

The implications of regional anesthesia after major surgery are multifaceted and not fully elucidated, thus requiring future studies with a focus on evaluating issues like implicating roles of mismanaged pain, environmental factors, stress responses associated with the perioperative period, long-term followups focusing on joint mobilisation and quality of life. 

This paper will elucidate the benefits of cPNB over intravenous analgesia and single-shot perineural blocks (sPNB) according to the published literature and show some aspects for possible future directions.

## 2. The Need for Optimal Postsurgical Analgesia

In western countries, 50–70% of in hospital patients suffer from moderate-to-severe pain after surgery [28], and 40% of ambulatory surgical patients have moderate/severe pain during the first 24–48 h [29]. Orthopedic surgery remains the major indication for peripheral nerve blocks for anesthesia and postoperative analgesia [30]. Orthopedic surgery is one of the most if not the most painful surgery [31, 32]. In fact, after orthopedic surgery significant increases in pain scores may persist for 2 to 3 days [33, 34]. In postoperative evaluations of hip and knee surgeries using questionnaires 28% of patients after total hip arthroplasty [35] and 33% of patients after total knee arthroplasty [36] suffered from chronic pain. The common finding between these patients was the intensity of immediate postoperative pain. Intense persistent postsurgical pain has indeed been described as a risk factor for the development of chronic pain [37, 38]. Therefore, an improvement in postsurgical pain therapy could be an important factor to reduce pain chronification and its adverse effects on health system costs [39] (Table 1).

## 3. cPNB Insertion Techniques

### 3.1. Skin Preparation and Patient Draping

**Figure 1:**
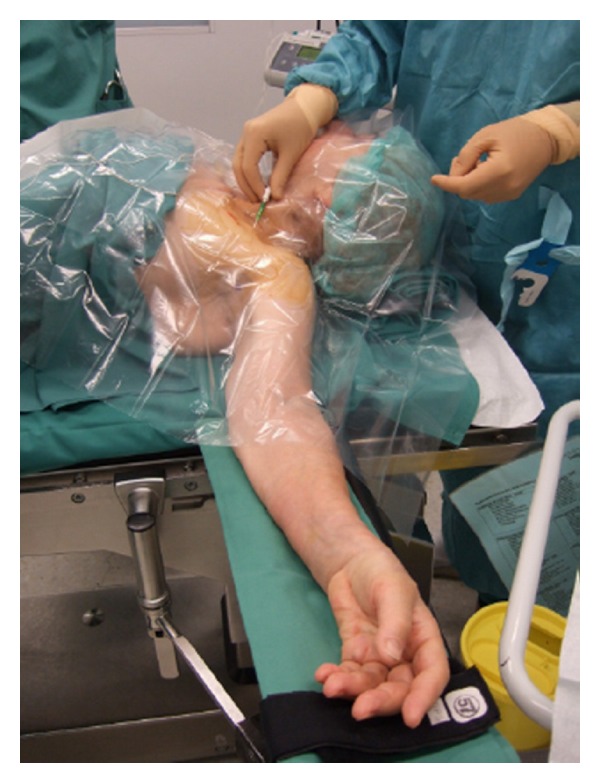
Sterile precautions for regional anesthesia.

**Figure 2:**
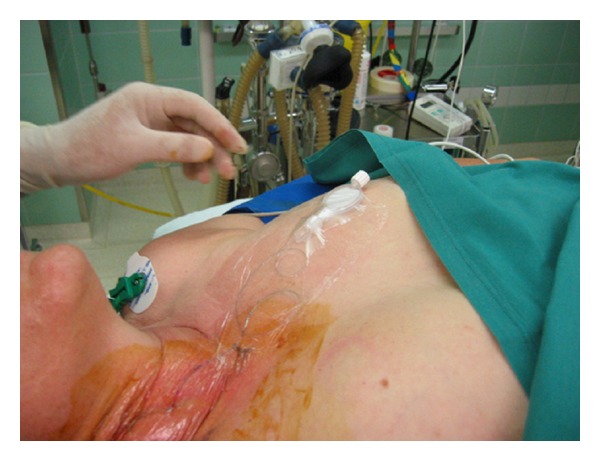
Sterile catheter dressing after catheter tunneling.

Sterility is of utmost importance for the performance of cPNB independently of the technique performed (neurostimulation or ultrasound). The American Society of Regional Anesthesia and Pain Medicine (ASRA) recommends antiseptic hand washing, sterile gloves, surgical mask and hats, and the use of alcohol-based chlorhexidine antiseptic solutions [40]. Further sterile protection of the ultrasound probe is essential [41]. The catheter dressing after insertion has to be sterile and transparent to avoid and recognize catheter infection (Figures 1 and 2).

### 3.2. Needle Choice

Short bevel needles are widely accepted as standard practice in regional anesthesia. The literature is not conclusive to support the notion that nerve injury is minimized using short bevel needles [42–44]. The use of ultrasound for catheter placement is increasing and apart from thin-walled needles also Tuohy needles remain popular with this technique. However, despite the use of ultrasound, the risk of intraepineurial local anesthetic injections may be quite common as described by Liu et al. in a large prospective study on ultrasound-guided interscalene and supraclavicular blockade for shoulder surgery [45] and different case reports [46, 47]. For fascia iliaca blocks a blunt needle (pencil point or Tuohy) offers a better recognition of a “pop” as tactile feedback when the different fascias are pierced [48].

### 3.3. Nerve Stimulation

Before introduction of portable nerve stimulators in 1962 [49], PNB were performed using induced paresthesia (“no paresthesia and no analgesia”), [50] fascial “pop,” [51] or even with fluoroscopic guidance [52]. Despite the fact that there has never been a repeated comparison of nerve stimulation and paresthesia which has been for a long time the most wide-spread technique and is still used, nerve stimulation has been considered the gold standard at least until the introduction of ultrasound in 1989 [53]. Using electrical current to place an insulated needle close to a peripheral nerve to inject local anesthetic or place a perineural catheter is the basic principle of nerve stimulation [54]. Without doubt, if properly used nerve stimulation offers a high success rate for sPNB and for cPNB [55–62]. The most important rules to achieve competitive success rates for sPNB are

- use a proper technique with logical (adaptable to all patients, independent of body height and weight) and reproducible landmarks [56–59];
- accept only responses with previously described high success rate (e.g., distal responses of the posterior cord for infraclavicular block; inversion for popliteal block) [56, 57, 63];
- inject only at following nerve stimulator setting (with still weak motor response visible): amplitude width: 0.1 ms, frequency: 2 Hz, and impulse: 0.3–0.4 mA;
- use the correct block for the planned surgery (e.g., not an interscalene block for elbow surgery [64] but an infraclavicular block) (Table 2).

Often comparative studies which described unacceptable low success rate for nerve stimulation are based on violation of at least one of the above-mentioned principles [64–67]. Although unclear data exists showing a consistent relationship between stimulating current and proximity to the nerve [68–70], the above-mentioned nerve stimulator setting is associated with high success and low complication rates [56].

**Figure 3:**
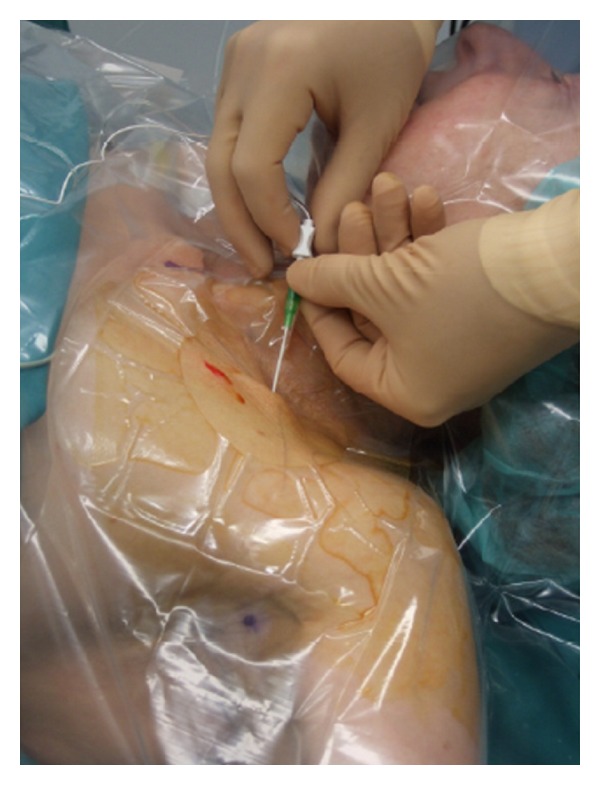
Cannula over the needle technique.

**Figure 4:**
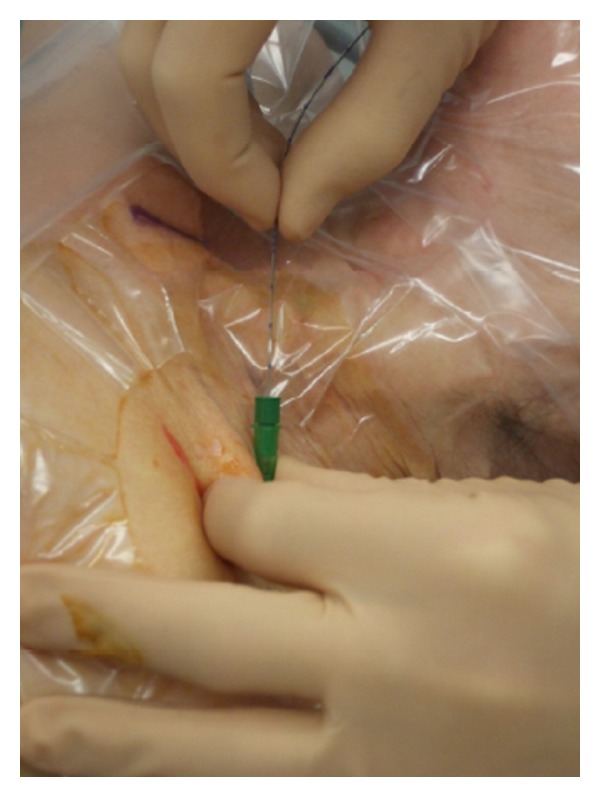
Catheter insertion through the cannula. Catheter is advanced up to 5 cm over the cannula tip.

**Figure 5:**
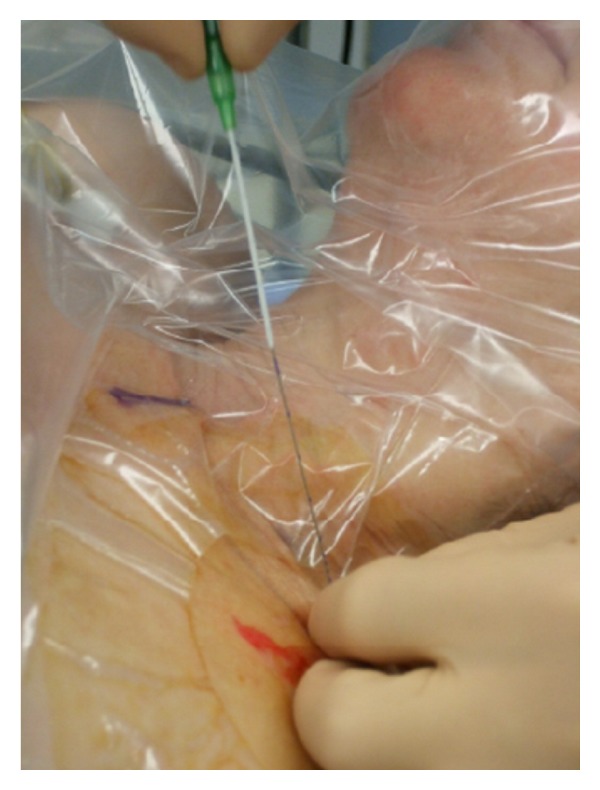
Cannula removal leaving the catheter in place.

**Figure 6:**
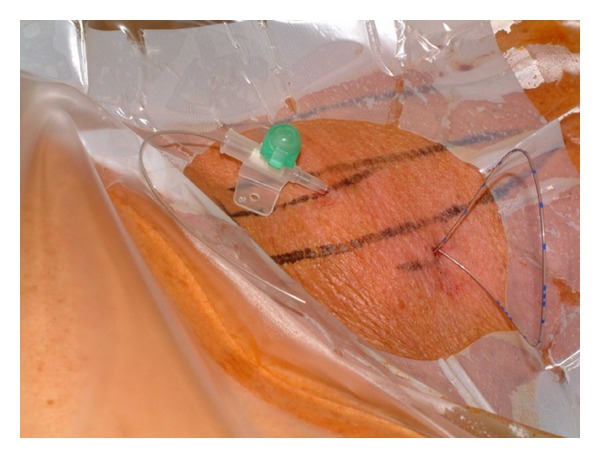
Catheter tunneling.

For catheter placement, it is of prime importance that the needle is guided tangentially to the nerve to avoid nerve injury and to guarantee a catheter placement parallel to the nerve (Figure 3). In this case, a 3-orifice catheter will facilitate a spread of the local anesthetic along the nerve compared with an end-orifice catheter. To avoid early catheter displacement, the insertion should be performed before injection of local anesthetic [74]. To our mind, for catheter placement the cannula over the needle technique [26] threading the perineural catheter 3-4 cm past the tip of the cannula with subsequent subcutaneous tunneling for 4-5 cm to avoid displacement after insertion is the technique of choice (Figures 3, 4, 5 and 6). The optimal distance to advance a perineural catheter past the needle tip remains unknown according to the literature, but data suggests that increasing the insertion distance >5 cm is correlated with an increased risk of catheter coiling [75]. As there is no data suggesting insertion lengths >5 cm, the maximal insertion depth should be considered 5 cm [75, 76].

An further option is the use of stimulating catheters: with an insulated needle electrical current is used to locate the target nerve, followed by the insertion of a perineural catheter that conducts current to its tip [77]. Although there is evidence supporting some advantages in certain anatomic locations (popliteal fossa, femoral, and interscalene region) [78–80], the clinical relevance considering the significantly increased material costs remains unclear [81–83].

### 3.4. Ultrasound

The introduction of ultrasound for regional anesthesia has been an important development. First it was considered a supplement to nerve stimulation, now it is recognized as a “stand-alone” technique [84]. Unfortunately, a nonending dispute between nerve stimulation and ultrasound has distracted the focus of interest from clinically important questions [85, 86]. Almost all comparative studies violate in a way or another at least one of the crucial points for correct nerve stimulation mentioned above and have been subject to criticism [87–89]. The recent review by Abrahams et al. [90] dealing with this topic emphasized the superiority of ultrasound according to the present “evidence” and was severely disputed in web publications of letters to the editor [91]. There are also some publications comparing ultrasound-guided catheter insertion versus nerve stimulation catheter insertion [92–95]. Actually, some of these studies report at least similar analgesia between both techniques [96–98]. Regrettably, in most of these publications the inappropriate use of neurostimulation lead to intolerable low success rates (<80%). Moreover, surrogate outcome like needle passes through the skin, needle time under the skin, patient satisfaction, local anesthetic volume, and other parameters of doubtful clinical importance have been introduced to compare these techniques. The comparison of ultrasound with stimulating catheters shows a similar picture: ultrasound is at least as effective with improvement of insertion-related discomfort and insertion time [93, 99]. The limited length and the main focus of this review preclude a further indepth discussion of this topic. Indeed, there are randomized controlled trials suggesting that stimulating catheters for popliteal blocks [100], and the combination of ultrasound and nerve stimulation for catheter insertion improve analgesia [101]. Both techniques have to be considered equal if properly used with the respective advantages and disadvantages. This statement is in accordance with the most recent recommendation of the ASRA [102].

Data from controlled trials involving ultrasound-guided sPNB is not automatically applicable to ultrasound-guided cPNB. However, many reports confirm the feasibility of ultrasound-guided catheter-insertion with a great success rate [134, 135] also if there are few RCTs available [19, 136–138]. The description of the different techniques for ultrasound-guided catheter insertion (needle out-of-plane with nerve in short-axis approach and needle in-plane with nerve in short-axis or long-axis approaches) are well described in a recent editorial [139].

The combination of nerve stimulation (insulated needle or stimulating catheter) and ultrasound guidance for catheter placement is controversially discussed in the literature, and often the difficulties are major compared to the single use of either technique [101, 140–142].

In certain situations ultrasound might be, at least theoretically, superior to nerve stimulation for example when sensory nerves are the target [143], after limb amputations [144] if there is no phantom sensation to guide nerve stimulation [145], when vascular puncture is an absolute contraindication like in anticoagulated patients [146], or when an electrically induced muscle response is impossible [147] or not desired [148]. However, ultrasound needle and catheter tip visualization are often difficult specially in deep structures. In these cases, nerve stimulation might prove beneficial [149]. 

## 4. Local Anesthetics, Infusion Rates, and Delivery Strategies 

### 4.1. Local Anesthetics & Adjuncts

The most commonly used local anesthetics for cPNB are bupivacaine and ropivacaine, and both seem to provide adequate analgesia without major toxicity. However, ropivacaine is known to be more “motor sparing” [55] and less cardiotoxic compared to bupivacaine [150]. However, cases of toxicity involving both bupivacaine and ropivacaine are almost exclusively related to a single large injection of local anesthetic. At the concentrations of 0.15% for bupivacaine and 0.2% for ropivacaine, they seem to provide similar analgesia with little difference in hand strength preservation [151, 152]. Recently, for interscalene cPNB after open rotator cuff repair ropivacaine 0.3% was compared to 0.2% showing a decrease in morphine consumption, a better sleep quality, and no impairment in hand strength [55]. 

To prolong the effect of local anesthetics, the addition of different drugs to local anesthetics has been suggested for sPNB and cPNB like opioids [165, 166], epinephrine [167], and clonidine [168], but their safety or clinical benefits like reduction of local anesthetic use for cPNB remain unclear [169, 170]. 

### 4.2. Infusion Rates and Delivery Strategies

Preliminary evidence does not suggest a rigid local anesthetic delivery system for all cPNB. According to the literature, total local anesthetic dose and not its concentration or its delivery rate mainly influences clinical effects, although this issue is currently disputed in the literature [55, 162, 171–176]. At the moment, insufficient information is available to base recommendations on the optimal basal rate, bolus volume, and lockout period for the different variables that may affect these values (catheter location, catheter type, surgical procedure, etc.). As a rule, we can state that basal infusion of local anesthetic reduces breakthrough pain and improves sleep quality [33, 173, 177]. The addition of patient-controlled bolus doses further improves analgesia allowing a reduction of the basal rate and further allowing a reduction of opioids and their related side effects [33]. However, the comparison of basal-bolus and basal-only techniques related to pain scores and patient satisfaction is controversially disputed in the literature [33, 34, 177]. 

Reducing the basal rate might reduce the risk of toxicity and the reduction of motor and sensory block could prevent the risk for patient falls [178]. 

Until prospectively collected data are available, possible dose recommendations based on randomized controlled studies and clinical experience are given in Table 3. Although the maximum recommended hourly total dose of all local anesthetics during perineural infusion is still unknown [179], a wide safety margin has been reported in numerous clinical trials [180, 181].

For the outpatient setting, many types of pumps are on the market: reusable and disposable, nonelectronic and electronic, basal-only and basal-bolus capable [22, 182]. Nonelectronic disposable infusion pumps are divided in elastomeric, positive-pressure (spring-powered and gas-pressure-powered), and negative-pressure (vacuum) pumps [183]. The model chosen has to be adapted to the needs of the patient and meet with the costs. In clinical trials comparing electronic-programmable pumps with elastomeric pumps, elastomeric pumps have been shown to offer similar postoperative analgesia for fewer technical problems and lower costs [184]. In clinical practice, they were easy to use and accurate in function [185].

## 5. Why cPNB Instead of sPNB?

### 5.1. Pain Therapy

Different case reports suggest beneficial effects of cPNB for different indications [12, 186, 187], but published RCTs include only postoperative patients and address mainly pain relief, which is considered to be the main indication for cPNB [21].

Although single-injection nerve blocks (sPNB) also provide excellent analgesia, cPNB increases the flexibility of both duration and density of local anesthetic effect depending on the chosen dose. SPNB offers a good pain therapy for up to 24 hours but for this duration a dense motor block and important sensory loss must be taken into account. In situations like trauma surgery, where compartment syndrome might be masked by these dense and long-lasting blocks, these properties are not wished by the surgeon. However, there is not enough evidence to support the thesis that patient-controlled analgesia with opioids or regional analgesia delay the diagnosis of compartment syndrome provided patients are adequately monitored [188, 189]. Continuous peripheral nerve blocks offer the possibility to adapt to the different needs by lowering the volume or the concentration of the local anesthetic [153, 190]. This flexibility reduces the need for a large initial bolus reducing the risk of systemic toxicity. Moreover, the reduction of dense motor and sensory blocks reduces the risk of falls and positioning injury [178, 191, 192]. An increase of local anesthetic concentration can also improve patient outcome. Borgeat et al. increased the ropivacaine concentration for interscalene blocks from 0.2 to 0.3% after rotator cuff repair and described a significant reduction of morphine consumption and a better sleep quality for the first postoperative night without increasing the intensity of motor block or side effects [55].

### 5.2. Upper Extremity

Most of cPNB benefits are mainly dependent on successfully improving pain control, reducing opioid consumption and its related side effects and increasing patient satisfaction [21]. Like for shoulder and elbow surgery, potent analgesia is achieved and maintained with cPNB due to their complete innervation by nerves affected by the perineural infusion [55, 58, 59].

For shoulder and upper arm surgery, the interscalene nerve block (ISNB) has become a standard procedure in specialized centers [193]. This technique has been evaluated in different RCTs using either neurostimulator or ultrasound and as has been shown to be superior to settings using subcutaneous/oral opioids or even opioid-based patient controlled analgesia (PCA) [27, 34, 58, 138, 153–155, 194–200]. The superiority of continuous interscalene block (cISNB) towards single-shot interscalene block (sISNB) was demonstrated for moderate painful and extremely painful surgery in different studies [153–155, 196, 200, 201].

The benefits for elbow surgery using continuous infraclavicular perineural block is well validated [202], but good analgesia needs a high dose of local anesthetic leading to insensate extremities [175]. However, for surgical procedures distal to the elbow, brachial plexus infusions seem to provide less impressive analgesia [202]. Moreover, benefits of axillary [203] and supraclavicular [204, 205] continuous infusions demonstrated by RCTs are still lacking. 

### 5.3. Lower Extremity

Femoral or posterior lumbar plexus infusions are well validated for hip [158, 187, 206, 207] and knee surgery in RCTs [208–210] but might result in dangerous quadriceps femoris and hip adductor weakness if high doses of local anesthetic are administered to optimize analgesia [178]. Moreover, contrary to the brachial plexus, in the lower extremity a single perineural infusion will not cover all surgical sites as these are innervated by multiple nerves. Therefore, a single perineural infusion might not provide optimal analgesia without the concurrent use of additional analgesics [171, 211]. A valid method to achieve optimal analgesia is the supplementation of a continuous perineural infusion with a separate single-injection peripheral nerve block as is often done for knee arthroplasty (continuous femoral nerve block complemented by a sciatic block) [212]. Inserting a second catheter for continuous perineural infusion has been suggested [26, 206, 213], but limited available data are conflicting and not useful to establish a clear clinical practice [26, 214]. Moreover, the recent review by Paul et al. [215] has brought new light into the clinical standard for pain management after total knee arthroplasty. Only two RCTs [211, 216] compared single-shot femoral nerve block to continuous infusion of local anesthetic according to his inclusion criteria. The additional use of single-shot sciatic nerve block was also investigated. According to the chosen methodology he suggested that at the moment, there is not enough evidence to support the use of neither a single-shot sciatic nerve block nor a continuous perineural femoral nerve block in addition to a single-injection femoral nerve block [217]. Despite the emotional reactions to this review [218] fearing that the use of cPNB will be questioned by surgeons according to the missing evidence, further studies should address this question to establish the correct approach for this surgery [212].

Also if lumbar epidural provides generally equivalent analgesia to femoral perineural infusion for both, hip and knee arthroplasty cPNB offers a more favorable side effect profile avoiding the risk of epidural hematoma during anticoagulant administration [25, 207]. Therefore, cPNB has to be considered the method of choice if regional anesthesia is indicated for the lower extremity. 

For foot and ankle surgery, the use of cPNB has clearly demonstrated its superiority to other analgesia regimens. It has been shown to decrease hospital costs and length of stay [219]. Several studies have shown that regional anesthesia for foot surgery is safe and does lead to reduced perioperative opioid requirements [56, 57, 220]. Patients have reported improved sleep, reduced pain scores, and faster recovery times [162, 190, 221–223]. Patient satisfaction scores improved after regional anesthesia compared with general anesthetic with following systemic analgesia [224–226]. Recently, Blumenthal et al. described, that an additional femoral nerve catheter with ropivacaine 0.2% to a continuous popliteal catheter with ropivacaine 0.2% both for 48 hours for major ankle surgery reduces pain during motion compared to popliteal catheter alone. This effect was still present after 6 months suggesting that short-duration regional anesthesia has a late impact on residual pain after major ankle surgery [26]. 

## 6. Functional Outcome

### 6.1. Short-Term Use of cPNB

The effects of the short-term use of cPNB on functional outcome after orthopedic surgery are still controversially discussed. Ilfeld et al. reported in a retrospective study that in the 3 days after a total shoulder arthroplasty, a continuous interscalene block using ropivacaine 0.2% was associated with increased range of shoulder motion due to the complete analgesia provided [227]. These results were supplemented by a following prospective study analyzing the effects of ambulatory continuous interscalene nerve block after shoulder arthroplasty [27]. Authors concluded that an ambulatory continuous interscalene nerve block considerably decreased the time until readiness for discharge (defined as achievement of a previously established range of motion and good pain control) after shoulder arthroplasty. This result was achieved primarily by providing potent analgesia permitting greater passive shoulder movement and by the avoidance of intravenous opioids. However, recently Hofmann-Kiefer et al. compared a continuous interscalene infusion of local anesthetic with an opioid-based PCA regimen. They reported that continuous interscalene block improved analgesia without improving postoperative function during early rehabilitation of the shoulder joint. [198]. For knee surgery, De Ruyter et al. reported that a continuous femoral nerve block provided satisfactory analgesia, improved rehabilitation, and reduced hospital length of stay compared with opiates [228]. Contrary, Raimer et al. described better analgesia with continuous psoas and sciatic blocks or epidural catheters compared to opioid PCA regarding pain levels, analgesic requirements, and patient satisfaction. However, there was no difference in functional outcome between the 3 groups [229]. 

### 6.2. Benefits after Catheter Removal

Despite the overwhelming evidence highlighting cPNB benefits during local anesthetic infusion, there exist few data demonstrating benefits after catheter removal. These data include improved analgesia a few days after removal [61, 186, 216] and after 6 months [26], faster resumption of unassisted standing [186], and improved passive knee flexion leading to earlier discharge from rehabilitation centers [25, 230]. The often cited increase in health-related quality of life has been shown only in 1 study [231] but could not be reproduced in many other more recent studies [232–236].

These results are in accordance with the review by Liu and Wu analyzing the effect of analgesic technique on postoperative patient-reported outcomes [20]. Authors found that regional anesthesia offered better postoperative analgesia control with reduction of opioid-related side effects, but there were insufficient and inconsistent data to support subsequent improvement in quality of life, quality of recovery, satisfaction, and length of hospital stay.

Therefore, more studies focusing on the medium- or long-term improvements in health-related quality-of-life measures are needed [234, 237]. 

### 6.3. Impact on Postoperative Cognitive Dysfunction

Postoperative cognitive dysfunction (POCD) is an important issue in an aging society. Postoperative cognitive dysfunction is a poorly defined condition as numerous studies use different definitions and neuropsychological tests to detect potentially altered short- or longer-term memory, motor control or information processing following anesthesia. There is evidence that altered intraoperative physiology may influence POCD and longer-term outcomes. A meta-analysis of 21 studies on POCD and postoperative delirium (POD) found no effect of anaesthesia type on the odds ratio of developing POD (0.88, 95% CI 0.51–1.51) [238]. However, it has been suggested that avoidance of general anesthesia and of central acting analgesics in the elderly leads to reduced postoperative long-term cognitive dysfunction [163]. The most recent study suggesting that anesthesia regimen has no impact on cognitive dysfunction had severe protocol inaccuracies, as the regional anesthesia group (spinal anesthesia) was premedicated with a benzodiazepine, another benzodiazepine was added prior to spinal anesthesia, a bispectral index <60 was achieved with propofol or with gas during surgery, and pain therapy was performed with oral oxycodone and subsequent morphine PCA [164]. Further well-designed studies are needed to assess the impact of continuous regional anesthesia for surgery and postoperative pain treatment on the incidence of POCD.

## 7. Ambulatory and Home Therapy

Apart from the classical postoperative setting, cPNB has been successfully introduced in the ambulatory surgery setting for both adults [134, 191] and pediatric patients [239]. 

Continuous peripheral nerve blocks offer the opportunity to deliver effective pain therapy at home and are considered to be a valid alternative to opioid-based analgesia and its related side effects [240]. However, a proper patient selection is essential for safe cPNB at home, as not all patients are willing to accept the extra responsibility of the catheter and pump system [191, 240].

Additionally, patients with known hepatic or renal insufficiency are excluded from ambulatory cPNB to avoid possible local anesthetic toxicity [241]. Obese patients and those with heart or lung disease who cannot compensate for mild hypercarbia and/or hypoxia are excluded from interscalene and cervical paravertebral infusions, which are known to affect the phrenic nerve and ipsilateral diaphragm function [155].

## 8. Economical Outcome

The advantages of sPNB over general anesthesia related to cost-effectiveness in the operation theatre have been recently described by Gonano et al. for shoulder arthroscopy [242]. Authors could show that ultrasound-guided interscalene blocks lead to an improvement of anesthesia-related workflow and to a reduction of postanesthesia care unit (PACU) time compared to general anesthesia. The positive effects of regional anesthesia on the anesthesia-related costs are well known: reduction of postoperative nausea and vomiting, reduced length of stay, successful same day discharge, reduction of unplanned admission or readmission, reduction of multiple-day hospitalizations to single days, earlier discharge, reduction or even elimination of PACU length of stay leading to reduced postoperative nursing interventions, faster discharge times, and reduction of operating room time without increase in turnover time [211, 243–245]. Fredrickson and Stewartcompared recently continuous interscalene nerve block for rotator cuff repair to combined single injection interscalene block with additional postoperative intermittent intra-articular local anaesthetic infiltration and to intermittent intra-articular only local anaesthetic infiltration. Authors concluded that continuous interscalene nerve block following rotator cuff repair in a multiprovider setting was associated with reduced total opioid/tramadol and antiemetic consumption, without a significant increase in the monetary costs [246].

Unfortunately, there are no studies comparing sPNB and cPNB regarding cost-effectiveness. However, the duration of hospitalization [247] or even the need for hospitalization [248] can be reduced by the use of cPNB reducing inpatient treatment costs.

## 9. Benefits of cPNBs

The impact of cPNBs on analgesia has been described in many RCTs and has been elucidated above [21]. The related dramatic decrease in required supplemental opioids, opioid-related side effects, and sleep disturbances, while simultaneously increasing patient satisfaction are further benefits [190]. Moreover, a decreased time to adequate ambulation with additional optimization of daily activities after ambulatory cPNB compared with intravenous opioids has been described [34]. For continuous regional anesthesia following shoulder and knee arthroplasty, an accelerated improvement of passive joint range of motion potentially leading to shorter hospitalization has been described [25, 27, 216, 230]. 

Ambulatory shoulder arthroplasty and 23-hour-stay knee and hip arthroplasty using ambulatory continuous interscalene, femoral, and psoas compartment nerve blocks, respectively have been reported [247, 249, 250]. 

However, although postknee arthroplasty inflammation is reduced after a continuous femoral nerve block [186]. the cPNB could not produce major improvements in long-term outcomes such as decreased chronic pain and improved health-related quality of life [20, 235, 251]. Studies focusing on long-term outcomes are lacking to evaluate the effects of cPNB after 6 and 12 months on costs, functional outcome and socioeconomic aspects.

## 10. Adverse Effects

Two prospective investigations involving more than 2.100 patients suggest that the incidence of cPNB-related complications is very low and comparable to sPNB techniques [58, 252]. However, different catheter insertion techniques, different anatomic locations, variations in equipment and different infusion regimens make comparisons difficult. In fact, several prospective studies report an incidence of secondary block (during infusion) failure of 1% [253], 20% [197], and 50% [254]. Therefore, all reported complications in this chapter cannot be translated to the different clinical practices.

### 10.1. Minor Complications

These complications are frequently seen in clinical practice and include catheter obstruction, catheter dislodgement, fluid leakage, disconnection from the pump system, infusion pump malfunction, and skin irritation or allergic reactions to the sterile catheter dressing [202, 252, 253, 255, 256].

### 10.2. cPNB-Specific Complications

These seldom but possible complications include inaccurate catheter tip placement resulting too far from the target nerve and therefore lacking successful analgesia, [74] or in an undesirable position like intravascular [257], intrapleural [258], intraneural [259], epidural [260] or even intrathecal. [261–263]. However, whether catheter migration is possible after correct placement remains unclear [264, 265].

### 10.3. Infections

Most studies reporting infection in relation with cPNB derive from hospitalized patients even if data for large outpatient series are increasing. Although the reported rates of inflammation (3%-4%) [86, 252, 253] and catheter bacterial colonization (6%–57%) are apparently high [266, 267], the incidence of catheter infection for inpatients ranges from 0% to 3.2% [252, 266, 268], and for outpatient rates are below 1% [191, 269]. 

Risk factors for infection in the setting of cPNB include lack of or inappropriate antibiotic prophylaxis, and axillary or femoral catheter location [269]. However, other studies have reported the interscalene location as the most problematic for infection [253, 268]. Further risk factors are admission to an intensive care unit, male sex [252], and increased infusion duration [252]. Though, some case reports demonstrate that prolonged catheter use for more than 30 days is possible without increased rate of infection [15]. There is limited evidence proving that subcutaneous catheter tunneling [270] may decrease the risk of bacterial colonization and infection. 

Although life-threatening catheter-related infections and sepsis have been reported in the literature [271, 272], no published case of permanent injury due to cPNB-related infection is reported in the literature [269]. 

Sterility precautions while filling the infusion pumps are of utmost importance. In a recent case report of a severe, deep cellulitis, evolving to mediastinitis was attributed to contaminated infusate from a pump which was manipulated in nonsterile conditions [272]. Therefore, anesthetists should act upon the recommendations of the American Society of Regional Anesthesia and Pain Medicine (ASRA) using sterile precautions such as antiseptic hand washing, sterile gloves, surgical masks and hats, and using alcohol-based chlorhexidine antiseptic solutions to avoid such complications [40].

### 10.4. Neurologic Complications

Although neurologic injury associated with cPNB is usually transient ranging from 0.3% to 2.0% [33], it remains the most feared concern performing regional anesthesia. Injury may occur during catheter placement or even in the postoperative period. For regional anesthesia in general the incidence of transient adverse neurologic symptoms associated with cPNB is 0% to 1.4% for interscalene [253, 273], 0.4% to 0.5% for femoral [252, 274], and 0% to 1.0% for sciatic catheters [266, 274]. Neuburger et al. described a 0.2% incidence of neurologic deficits lasting more than 6 weeks in nearly 3.500 catheters from multiple anatomic locations [253]. However, it remains unknown whether the deficits resolved spontaneously after the 6-week study period. Other prospective investigations report that the overwhelming majority of neurologic symptoms still present at 4 to 6 weeks resolve spontaneously within 3 months of surgery [252, 273]. 

There are also cases of long-term or permanent nerve injury in patients after perineural infusion [275]. In five large, prospective series with patient followups at least three months after, 3 cases of unresolved adverse neurologic events were found [58, 252, 266, 273, 274]. The combination of these results including 4.148 patients suggests that the risk of neurologic injury lasting longer than 9 months associated with cPNB is 0.07%. The role of the continuous infusion for the evolution of these complications remains unclear. 

Only few reports of nerve injury in patients sent home with cPNB are described in the current literature. However, pressure injury due to insensate extremity has been implicated as a likely cause [10]. Therefore, particular precautions must be taken when casts or splints are placed on patients with insensate extremities. 

A further important neurologic concern associated with in- and out-patient cPNB is the risk of falls. Williams et al. reported recently 1.7% of outpatients treated with continuous femoral nerve block fell due to insensate lower extremity despite adequate instructions how to behave at home [192]. Ilfeld et al. pooled data from 3 previously published, randomized, triple-masked, and placebo-controlled studies of cPNB involving the femoral nerve after knee and hip arthroplasty and demonstrated a causal relationship between cPNB and the risk of falling after these surgical interventions [178]. 

This complication elucidates the potential benefit in providing low-concentration blocks that preserve more motor function and proprioception. Moreover, the desire to offer complete analgesia should be balanced against the risk of falling. Therefore, the recently published study dealing with multimodal analgesic approaches including regional anesthesia can offer a solution to achieve excellent analgesia while preserving motor function [276].

### 10.5. Local Anesthetic Toxicity

Most investigators report basal hourly infusion rates of 5 to 10 mL/h using dilute solutions of either ropivacaine or bupivacaine [33, 34]. Despite these low rates of infusion, local anesthetic toxicity is reported as a possible complication of cPNB [277]. Systemic local anesthetic toxicity is a serious but rare complication using cPNB [278, 279]. Although, continuous infusion is unlikely to result in sudden onset of toxicity, patients treated with a pump with bolus capability are at risk if intravascular migration should occur. A further rare complication is myonecrosis after repeated large boluses of bupivacaine [280]. Also if there is in vitro and animal evidence for local anesthetic-caused neurotoxicity [281, 282], there seems to be little evidence that the risk of nerve injury from prolonged local anesthetic exposure might be increased in patients suffering diabetes or preexisting neuropathies [283, 284].

## 11. Future Directions

Local anesthetics act on the voltage-gated sodium channels [285]. The Na_v1.7_is the main channel for pain transmission in the peripheral nerves [286, 287]. Selective blocking of this channel for the postoperative period could be of special interest for ambulant cPNB avoiding the risk of falls. A high selective Na_v1.7_ local anesthetic would increase the ambulant use of cPNB with probably a remarkable impact in health costs.

Liposomes as reservoirs for drugs are biocompatible due to their biodegradability and low toxicity [288]. They can reduce the exposure of sensitive tissues like nerves to potentially toxic drugs like high concentrations of local anesthetics. Their administration is independent of technical skills required for catheter placement as a variety of routes are possible (topical, intramuscular, nasal, subcutaneous, oral, pulmonary, and intravenous) [289]. Even though not yet approved for peripheral nerve blocks, liposomal formulations of various local anesthetics offer an increase in clinical efficacy compared with the plain drugs [290]. Actual limitations of encapsulated local anesthetics are myotoxicity, neurotoxicity, tachyphylaxis, viscosity, and motor block [291]. Clinical studies describe a prolonged analgesia after infiltration and epidural application [292, 293]. Once the potential adverse effects like a prolonged motor block and toxicity are eliminated, these promising clinical results might be transferred to peripheral regional anesthesia. 

## 12. Conclusions

The Literature provides a plethora of information involving cPNB, but some aspects of perineural infusion remain controversial: the optimal catheter insertion modality, the optimal technique for each indication, the infusates offering the best safety, standardized local anesthetic delivery regimens, and optimization of continuous ambulatory infusion to reduce possible risks (as falling). The optimal analgesic technique for many surgical procedures has to be further elucidated, and cPNB must be compared with possible new analgesic techniques/regimens [231]. 

The new application areas for local anesthetic such as anti-inflammatory effects/anticancer effects [294, 295] have to be further investigated, and cPNB must be included in clinical trials addressing these topics. 

The socioeconomical aspect of anesthesia remains an important issue, and the role of cPNB for in hospital and at home use and their effects in the healing process and the readmission of the patients at work after surgery must be clarified. 

Prospective research addressing the above-mentioned issues can maximize the potential benefits and minimize the potential risks of cPNBs. 

## Figures and Tables

**Table 1:** Incidence of persistent postsurgical pain and associated disability. Based on data from [37, 71–73].

Surgery	Incidence	Severe disability
Amputation	30–85%	5–10%
Thoracotomy	5–67%	10%
Inguinal hernia repair	10–30%	2–4%
Breast surgery	11–65%	5–10%
Coronary artery bypass graft	30–50%	5–10%
Cesarean section	10–18%	4%

**Table 2:** Suggested catheter insertions for different surgical procedures. First choice is the first mentioned. The used literature for interscalene [58, 103, 104], cervical paravertebral [105–109], infraclavicular [59, 110–113], supraclavicular [114–116], axillary block [117–119], psoas compartment [120, 121], fascia iliaca [122–124], femoral [125–127], obturator [128, 129], popliteal sciatic [56, 57], and proximal sciatic [130–133].

Surgery	Catheter insertion location
Shoulder / proximal humerus	Interscalene; cervical paravertebral
Distal humerus, elbow, forearm, and hand	Infraclavicular; supraclavicular; axillary block
Hip	Psoas compartment; fascia iliaca; femoral
Thigh / knee	Femoral; fascia iliaca; obturator; proximal sciatic
Calf / ankle / foot	Sciatic (popliteal; proximal); add femoral nerve block for major ankle surgery [26]

**Table 3:** Recommended doses of different local anesthetics for different catheter locations and their administration regimen according to clinical practice of the authors (CP), own publications or based on selected randomized controlled trials. Ropi: ropivacaine; Bupi: bupivacaine; B: basal rate (ml/h); Bo: bolus (ml); L: lockout (min). The used literature for Interscalene [55, 58, 153–157], infraclavicular [33, 59], axillary, [7] femoral [158–160], Fascia iliaca [161], subgluteal sciatic [162], and popliteal sciatic [51, 52, 78, 163, 164].

Catheter location	Local anesthetic	Infusion rate
Interscalene	(i) Ropi 0.2%	(i) CP: B: 4–6; Bo: 4–6; L: 20–30
	(ii) Ropi 0.3%	(ii) CP: B: 3–5; Bo: 3-4; L: 20–30
	(iii) Bupi 0.125% (sufentanil 0.1 *μ*g/ml and clonidine 1 *μ*g/ml)	(ii) CP: B: 5; Bo: 2.5; L: 30

Infraclavicular	(i) Ropi 0.2%	(i) CP: B: 4–6; Bo:4–6; L: 20–60

Axillary	(i) Bupi 0.25%	(i) B: 10 / B: 0; Bo: 10; L: 60

Femoral (i) Hip surgery (ii) Knee surgery	(i) Hip	(i) Hip
	(a) Ropi 0.2%	(a) B: 6; Bo: 4; L: 30
	(b) Bupi 0.125% (+sufentanil 0.1 *μ*g/ml and clonidine 1 *μ*g/ml)	(b) B: 10 / B: 0; Bo: 10; L: 60 / B: 0; Bo 5; L: 30
	(ii) Knee	(ii) Knee
	(a) Ropi 0.2%	(a) CP: B: 3–6; Bo: 2–4; L: 20–30 min
	(b) Bupi 0.125% (clonidine 1 *μ*g/ml)	(b) B: 5; Bo: 2.5; L: 30

Fascia iliaca (knee surgery)	(i) Ropi 0.2%	(i) B: 5; Bo: 5; L: 60 / B: 0; Bo: 10; L: 60

Subgluteal sciatic	(i) Ropi 0.2%	(i) B: 5; Bo: 5; L: 60

Popliteal sciatic	(i) Ropi 0.2%	(i) CP: B:4–6; Bo: 4–6; L: 20
	(ii) Levobupi 0.125%	(ii) B: 5; Bo: 3; L:15
